# Editorial: Bone health and development in children and adolescents

**DOI:** 10.3389/fendo.2022.1101403

**Published:** 2022-12-12

**Authors:** Federico Baronio, Fátima Baptista

**Affiliations:** ^1^ Department Hospital of Woman and Child, IRCCS Azienda Ospedaliero-Universitaria di Bologna, Bologna, Italy; ^2^ Department of Sport and Health, CIPER - Centro Interdisciplinar do Estudo da Performance Humana, Faculdade de Motricidade Humana, Universidade de Lisboa, Lisbon, Portugal

**Keywords:** androgen, diet, hypophosphatemia, puberty, perfluoroalkyl, obesity, osteogenesis imperfecta, vitamin D

Bone health is understood as the bone’s resistance to fracture, determined by the evaluation of bone mineral reserve, expressed as bone mineral content (BMC) or density (BMD). However, in a broader sense, bone health includes also the mechanical (support, movement, and protection) and extraskeletal (bone-derived factors) function of the skeleton (1). This Research Topic highlights several determinants of bone health (environmental, genetic, hormonal, nutritional, and mechanical) and their impact in children and adolescents.

During the growth period, the skeleton is constantly undergoing changes that go through bone modeling and remodeling, a process of bone strength optimization. Bone strength is especially “tested” during growth and aging as the incidence of fractures is higher in these periods of life (1, 2). Obesity seems to be a risk factor for bone fractures (3, 4). Despite a greater mechanical load associated with overweight and obesity, bone tissue is negatively influenced by the inflammatory state caused by cytokines released from adipose tissue (5–7). In general, overweight/obese children and adolescents have equal or higher bone mineral mass, but equal or lower cortical bone thickness than their normal body weight peers, at least in the forearm, which is the region where fractures are more frequent at these ages (6, 8–13). In this Research Topic, two studies with data from the National Health and Nutrition Examination Survey (NHANES) of the United States reveal a positive correlation between body mass index (BMI) and whole-body BMD, with a saturation effect. Both studies, conducted by Ouyang et al. and Wang et al. observed the existence of an optimal (and healthy) BMI for bone health. Another study analyzed whether serum levels of bone turnover markers (BTMs) are reduced or elevated in obese children. Cao et al. found that BTMs are reduced in overweight/obese children with significant differences according to age, sex, and pubertal stage that warrants further evaluations.

Peak bone mass - the maximum amount of bone mass reached between the second and third decade of life, is critical for bone strength. Proia et al. emphasized the need to investigate the triangulation between nutritional, endocrine, and mechanical (in this case physical activity) factors in children and adolescents’ bone tissue since most studies on this interaction were performed in adults.

The assessment of 25-hydroxyvitamin D3 serum levels should be part of the screening tests that pediatricians request for their at-risk patients to evaluate their calcium/phosphate metabolism. Xu et al. explored how in children with short stature the dosage of 25-hydroxyvitamin D2 [25 (OH) D2] and 25-hydroxyvitamin D3 [25 (OH) D3] alone could overestimate vitamin D stores. They showed that C3-epi/25(OH)D3 and 25(OH)D2/25(OH)D3 ratios determined from high-performance liquid chromatography-tandem mass spectrometry (LC-MS/MS) are more precise indicators of vitamin D reserves. The results, if confirmed in larger studies, suggest the use of LC-MS/MS as a more accurate laboratory technique for the evaluation of vitamin D status in short children.

Many genetic diseases affect bone, where osteogenesis imperfecta is one of the most serious conditions. Zhang et al. evaluated the skeletal outcomes of bisphosphonates treatment in a cohort of patients affected by osteogenesis imperfecta in which the duration of treatment was dependent on the achievement of age- and sex-specific BMD (~4 years). Overall, during three years of drug holiday, the authors showed a maintenance of BMD and fracture incidence; it was also observed a greater likelihood of restarting bisphosphonates treatment in patients with severe disease and in cases who started bisphosphonates treatment later in life.

Mindler et al. reviewed the radiological and clinical data of 43 patients with X-linked hypophosphatemia (XLH). The authors performed an analysis of the patients’ gait comparing the results with 76 healthy controls. They verified the negative impact of bone deformities and BMI on the quality of gait in patients with XLH.

Among 405 cases of children with finger problems treated in a hospital setting, Hao et al. described the eight cases affected by clinodactyly due to osteochondroma. In these patients, the surgical removal of the osteochondroma resulted decisive for the cure of clinodactyly although the procedure is not without complications and requires prolonged follow-up. A level of phalanx angulation greater than 10° should be investigated by hand radiography which is also useful for the preoperative characterization of osteochondroma.

Bone health is largely dependent on a proper balance of sex hormones. In particular estrogens deficiency in both adolescent girls and boys limits the maximization of peak bone mass in adulthood (14). Misakian et al. draw attention to the degree of insufficient bone mineralization in ~16-year-old adolescents with complete androgen insensitivity syndrome (CAIS), and showed that it may be even more severe in early gonadectomized cases. The authors observed that hormone replacement therapy did not lead to an optimal BMD in most of their patients, and pointed some explanations.

Classically, precocious puberty is treated with GnRH analogs, a well-established, effective, and safe therapy (15). In addition to the arrest of pubertal maturation, a slowdown in growth rate is among the treatment effects and it is caused by the interruption of bone growth plate development. Using animal models, Zhu et al. described the anabolic effect of stanazolol on the bone growth plate providing insight into the pathophysiology and rationale for its use during long-term GnRH treatment in cases with impaired growth velocity. Stanazolol has been safely utilized in Turner’s syndrome to increase final stature (16).

Two studies examined the impact that environmental interferents can have on bone health using data from the US (NHANES, 2005-2010). In one of the studies, Cui et al. found negative associations between blood lead levels and BMD of the lumbar spine, proximal femur, and femoral neck in boys and girls aged 8-19 years. Negative associations were greater in the spine than in the femur and greater in girls than in boys, suggesting that further studies on this topic are needed. Utilizing 1228 participants 12-19 years old from the same cohort, Xiong et al. found a negative correlation between the serum concentrations of several perfluoroalkyl substances and BMD. Again, these associations were more pronounced in the lumbar spine, in girls but also in those who were overweight/obese and had anemia.

Overall, this Research Topic renews awareness of several factors and mechanisms that affect bone health. We believe that the information provided reinforces the commitment of general health professionals, pediatricians in particular, in optimizing the growth and development of children and young people. Greater caution is needed to optimize bone health among the most vulnerable individuals, particularly those with medical conditions and those most exposed to health-threatening environments and lifestyles.

## Author contributions

FeB, FáB equally contributed to conception, design, writing and revision of the manuscript. All authors contributed to manuscript revision, read, and approved the submitted version.
